# The Gut Ecosystem: A Critical Player in Stroke

**DOI:** 10.1007/s12017-020-08633-z

**Published:** 2020-11-18

**Authors:** Rosa Delgado Jiménez, Corinne Benakis

**Affiliations:** Institute for Stroke and Dementia Research, University Hospital, LMU Munich, Feodor-Lynen-Straße 17, 81377 Munich, Germany

**Keywords:** Stroke, Microbiome, Metabolites, Gut-brain axis, Immune system

## Abstract

The intestinal microbiome is emerging as a critical factor in health and disease. The microbes, although spatially restricted to the gut, are communicating and modulating the function of distant organs such as the brain. Stroke and other neurological disorders are associated with a disrupted microbiota. In turn, stroke-induced dysbiosis has a major impact on the disease outcome by modulating the immune response. In this review, we present current knowledge on the role of the gut microbiome in stroke, one of the most devastating brain disorders worldwide with very limited therapeutic options, and we discuss novel insights into the gut-immune-brain axis after an ischemic insult. Understanding the nature of the gut bacteria-brain crosstalk may lead to microbiome-based therapeutic approaches that can improve patient recovery.

## Introduction

From the moment of birth, a symbiotic relationship is established between the host and the colonizing microbes in the gastrointestinal (GI) tract. These intestinal microorganisms regulate many aspects of the host physiology, such as metabolism, immune system education, and brain development (Sampson and Mazmanian 2015). Depletion of the microbiome in newborn mice results in impairment of the blood brain barrier, alterations in synaptic plasticity, and learning deficits (Sharon et al. 2016). Interestingly, all these developmental deficiencies are associated to an immature microglial phenotype, suggesting a key role of the gut microbiota in regulating brain development via its immunomodulatory properties (Pronovost and Hsiao 2019). Signaling between the gut microbiota and peripheral organs is mediated by microbiome-associated molecular patterns (MAMP) and microbiome-secreted metabolites that can either interact with the mucosal epithelial and intestinal immune cells, stimulate the vagus nerve or reach systemic circulation to signal to the brain and possibly modulate neuronal and immune response (Schroeder and Bäckhed 2016). In turn, parasympathetic and sympathetic nerve fibers that innervate the gut wall transmit inputs from the brain to influence gut motility, immune cell activity, and can induce changes in the gut composition (Furness 2012).

Recent findings have implicated the bi-directional communication between the gut and the central nervous system (CNS) in the onset and progression of brain diseases (Cryan et al. 2019). A number of neurological disorders and brain pathologies are accompanied by an altered microbiota composition and reduced gut motility (Hsiao et al. 2013). However, mechanisms underpinning gut microbiota and host crosstalk remain poorly understood. In this review, we focus on the role of the microbiome in ischemic stroke, the most prevalent type that accounts for approximately 80% of all strokes, and we discuss recent findings that had shed light on the mechanisms underlying gut-immune-brain crosstalk.

## Stroke Alters Microbiota Composition

The gut microbiota consists of different bacteria, viruses, and fungi that coexist in a synergic balance. Since the bacterial population in the intestine is better characterized, the majority of studies investigating the microbiome in health and disease targeted the bacterial communities. The gut microbiota is predominantly composed of four main bacterial phyla: Bacteroidetes, Firmicutes, Actinobacteria, and Verrucomicrobia, and disruption of the microbiota homeostasis confers disease susceptibility to the host (Arumugam et al. 2011).

Mounting evidences in experimental and clinical studies suggest that stroke alters the gut microbiota composition. Analysis of the gut microbiota of stroke patients revealed a shift in microbial composition in comparison to healthy individuals (Yamashiro et al. 2017) with the degree of dysbiosis correlated with the severity of the lesion (Haak et al. 2020; Yin et al. 2015). Similarly, the change in fecal taxonomic abundance is more pronounced in mice subjected to a severe than a minor stroke (Singh et al. 2016). However, when analyzing bacterial diversity and specific bacteria taxa, some discrepancies are observed across clinical and experimental studies. Indeed, studies reported an increased in diversity in stool samples from stroke patients in comparison to asymptomatic controls (Yin et al. 2015) or no change in microbiota diversity between sham operated and stroke mice (Stanley et al. 2016). In contrary, others observed a reduction in diversity with depletion of certain bacteria and overgrowth of others in both experimental and clinical settings (Yamashiro et al. 2017; Haak et al. 2020; Singh et al. 2016) suggesting that the degree of diversity could not necessarily predict for the severity of the stroke. Different studies observed an overall reduction of the Firmicutes, with a concomitant overgrowth of Bacteroidetes (Singh et al. 2016; Spychala et al. 2018). However, when analyzing microbiota changes at lower taxonomic ranks, there is a lack of consistency regarding the specific bacterial changes in clinical and experimental stroke (Benakis et al. 2020a, b). Differences in the location of fecal sampling (across the GI tract in mice, and stool samples in patients), the methods used for the extraction of genomic DNA and for 16S rRNA gene analysis, the severity of the stroke model and the baseline differences in the microbiota composition (the mouse origin or inter- and intra-individual variabilities in humans) could be confounding factors accounting for the contradictory results (Costello et al. 2009; Sadler et al. 2017; Stanley et al. 2018). Another confounding factor affecting the gut microbiota composition is the type of diet. The severe stroke cases are always hospitalized, where they will be treated with medicines and fed a hospital-controlled diet whereas little intervention occurs in mice after experimental stroke, which altogether will affect the microbiota composition in a different manner between rodents and humans. Overall, clinical and experimental data indicate a shift of the microbiota composition after stroke. However, to date, it is still not known which types of gut bacteria participate to the pathobiology mechanisms of stroke.

Aging alters the gut microbiota composition and is associated with an increased inflammation and intestinal permeability (Spychala et al. 2018)*.* Following stroke, mice harboring an aged microbiota had higher mortality, impaired locomotor function, and a pro-inflammatory cytokine response than young mice. The stroke outcome could be reverted in both young and old mice by fecal microbiota transplantation (FMT). These experiments highlighted the importance of age on the microbiota state and its impact on disease development, and how manipulation of the microbiota can lead to non-invasive therapies to both prevent and ameliorate stroke outcome. Whereas experimental findings indicate a correlation with age, gut dysbiosis and stroke outcome, the impact of other risk factors (diabetes mellitus, high blood cholesterol levels, alcohol consumption, high-fat diet, lack of exercise) and sex differences on the gut microbiome and stroke outcome remains to be addressed in experimental and clinical studies.

In experimental research, the mechanisms by which brain damage is thought to alter microbiome composition are by reducing gut motility mucosal barrier integrity due to post-stroke dysregulation of the autonomous nervous system (ANS) (Houlden et al. 2016). Following brain injury, there is a loss of cholinergic activity and an increase of adrenergic signaling in the gut which is associated with the disruption of the gut barrier. Accordingly, inhibition of β-adrenergic activity using beta-blockers significantly restored stroke-induced gut permeability and reduced bacterial dissemination to peripheral organs (Stanley et al. 2016). In this line, Singh et al. demonstrated that paralysis of the ileum alone can drive changes in the microbiota, arguing for a CNS-mediated dysbiosis (Singh et al. 2016). Gut bacteria can sense hormones and neurotransmitters derived from the GI tract, and some opportunistic pathogens are able to proliferate more in the presence of the stress hormone norepinephrine (NE) in in vitro culture (O'Donnell et al. 2006). Interestingly, following experimental stroke, there is an increased of NE release in the cecum, region of the gut between the small intestine and the colon, which could account for the proliferation of certain pathogens or commensal overgrowth over others (Houlden et al. 2016). In addition, the ANS modulates intestinal mucus secretion, and perturbation of the brain to gut signals is likely to have important effects on the size and quality of the intestinal mucus layer. In this respect, Houlden et al. observed a dysregulation of the mucoprotein production and a decrease of the mucus-producing goblet cells in the cecum after stroke (Houlden et al. 2016). Impairment of the formation of the mucus layer following stroke might allow commensals to come in direct contact with the epithelium, translocate and trigger an immune response, and post-stroke infection (Crapser et al. 2016; Stanley et al. 2016; Winek et al. 2016). Also more data regarding the direct implication of bacterial translocation leading to infection in experimental stroke are needed (Oyama et al. 2018). In conclusion, brain injury triggers an imbalance of cholinergic and adrenergic signaling in the gut, increases the concentration of released NE, reduces gut motility and alters the gut-brain barrier which are associated with microbiota dysbiosis. All these events will impact the gut microbial functional output and may further perturbs intestinal immune homeostasis.

## The Gut-Immune-Brain Axis in Stroke

Studies using animal models have provided the strongest evidence to date and have helped to elucidate the mechanisms underlying the gut-immune-brain interactions after stroke.

T lymphocytes are known to play a crucial role in the secondary tissue damage that follows brain injury. Pro-inflammatory T helper 1 cells (Th1), T helper 17 cells (Th17), and γδT-IL-17 + cells have been associated to contribute to post-stroke neurotoxicity, whereas regulatory T-cells (Treg) are known to exert anti-inflammatory and neuroprotective properties (Liesz et al. 2009; Shichita et al. 2009). There is a growing body of evidence supporting the key role of microbiota in maintaining the immune homeostasis by regulating the balance between pro-inflammatory Th17 and anti-inflammatory Treg cells in the GI tract.

**Fig. 1:**
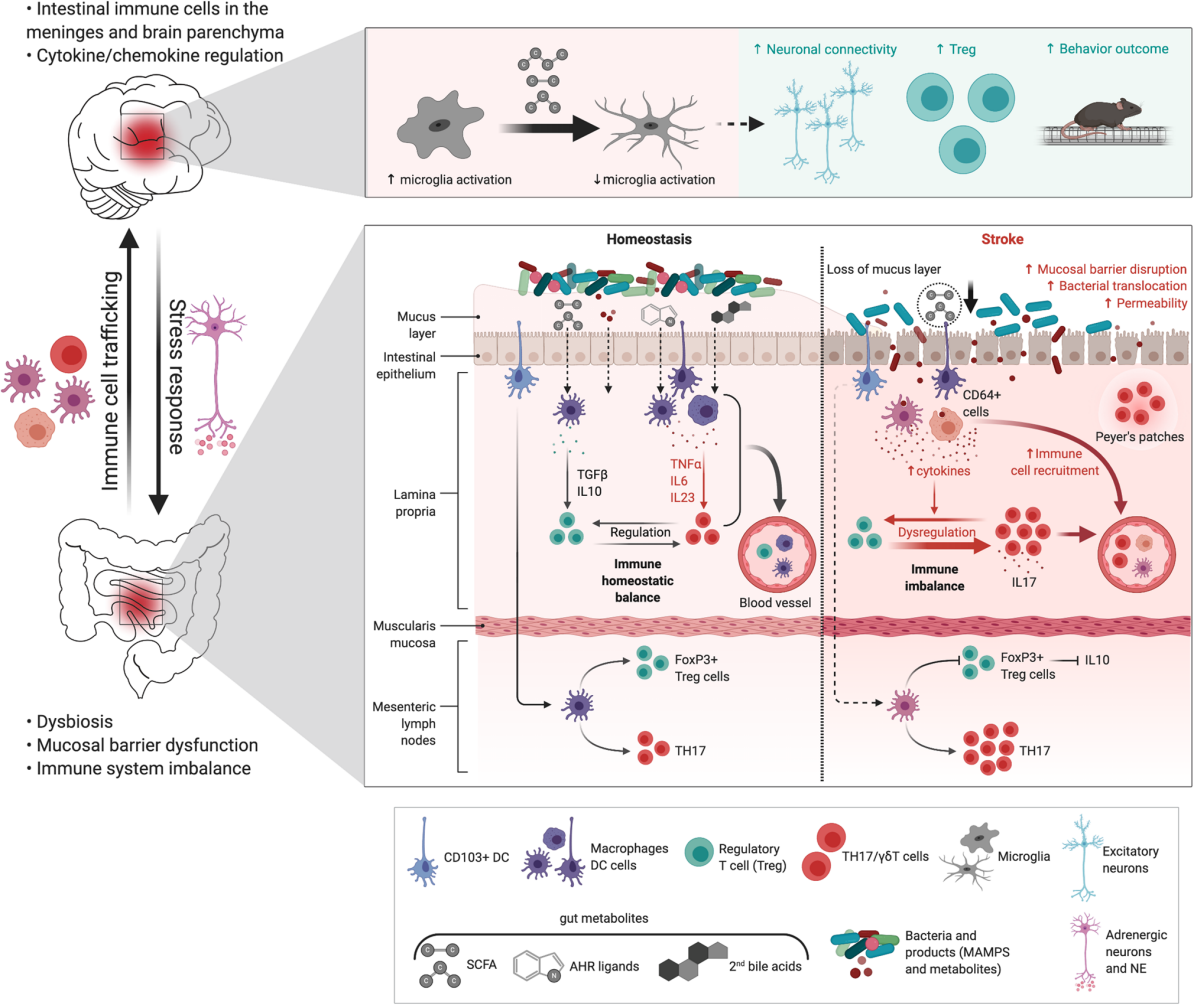
Gut metabolites–immune system crosstalk in stroke. Immune system regulation by the gut microbiome after stroke. Lower panel: In the gut, stroke induces dysbiosis, mucosal barrier dysfunction, an increase of gut permeability, bacteria translocation, post-stroke infection, and a pro-inflammatory T-cell response via dendritic cells (DC). Immune cells, especially T-cells, CD64 + macrophages and DCs migrate from the gut to the meninges and the brain after stroke. The role of gut metabolites (AHR, secondary bile acids, MAMPs) as immunomodulators of intestinal immune cells is not well defined yet. Microbiota-derived SCFA are decreased after stroke and possibly trigger an imbalance of γδT-IL-17 + cells and regulatory T-cells (Treg). Top panel: Supplementation of SCFA modulates neuronal activity and synapse density, and is associated with a decrease in microglia activation and an increase in Tregs together with a better recovery after stroke. Created with BioRender.com

Colonization of germ-free (GF) mice with post-stroke fecal content resulted in aggravation of the stroke outcome by inducing a pro-inflammatory Th1 and Th17-mediated response (Singh et al. 2016), suggesting that stroke-induced dysbiosis triggers a pro-inflammatory immune response which enhances brain damage. The authors demonstrated that T-cells coming from the Peyer’s patches invade the peri-infarct tissue and contribute to the immune response in the acute phase of stroke. Moreover, restoring gut microbiota homoeostasis with FMT from healthy donors reduced lesion size and increased the number of Treg cells in the ischemic brain (Singh et al. 2016). However, the sole presence of microbiota is neuroprotective as completely eradication of gut bacteria in GF mice resulted in greater infarct volume compare to colonized mice (Singh et al. 2018). Again, this microbiota-mediated beneficial effect on stroke outcome was dependent on the lymphocyte response (Singh et al. 2018), indicating that gut bacteria immune-modulatory role is essential for stroke recovery. The critical involvement of the gut-brain bi-directional communication in stroke was evidenced by showing that manipulation of the microbiota composition with antibiotics before the ischemic insult reduced the infarct volume and improved sensorimotor functions (Benakis et al. 2016). In this study, they showed that the neuroprotective effect is mediated by a microbiota-dependent priming of intestinal dendritic cells (DC), inducing an expansion of Tregs in the small intestine that act by suppressing the pro-inflammatory γδT-IL-17 + cells. Moreover, they identified IL-10 as a main orchestrator of Treg cell-mediated inhibition of γδT cell proliferation, since *IL-10 −/− *mice were exempt from the protective effects. This anti-inflammatory environment in the gut is relayed to the brain since fewer pro-inflammatory IL-17 + γδT-cells accumulated in the meninges, which is associated with smaller infarct volume. Interestingly, intestinal T-cells migrate from the gut to the meninges and the brain parenchyma demonstrating a direct gut-brain communication route in stroke (Benakis et al. 2016; Singh et al. 2016). These studies illustrate that microbiota is affected after stroke and that changes in the bacterial population promote a pro-inflammatory T-cell response, migration of intestinal immune cells to the meninges after stroke which altogether might participate in secondary brain damage and worsens stroke outcome. In addition, modification of the gut microbiota composition can skew the intestinal immune response towards an anti-inflammatory milieu via tolerogenic DC. Taken together, these findings highlighted a direct connection along the gut-brain axis via intestinal lymphocytes trafficking from the gut to the brain where they modulate the neuroinflammatory response to stroke (Benakis et al. 2016; Singh et al. 2016). However, it is still not known whether intestinal immune cells contribute directly to brain injury or repair mechanisms. Importantly, the molecular pathways underlying this gut bacteria-DC interaction remain elusive (Fig. 1).

## Manipulation of the Gut Microbiota in Stroke

Benakis et al. demonstrated that treatment of mice with ampicillin or vancomycin prior inducing the ischemic insult confers neuroprotection, whereas mice were not protected from stroke when using neomycin (Benakis et al. 2020a, b). This neuroprotection is associated with an antibiotic-specific shift in the microbiome composition with an expansion of Proteobacteria and Firmicutes, and a reduction of Bacteroidetes. Moreover, when they analyzed the predictive enzymatic pathways associated with the beneficial microbiota changes, they found that xenobiotic/aromatic compound metabolism was predictive of the size of the ischemic lesion (Benakis et al. 2020a, b; Stanley et al. 2018). Microbiota-derived metabolites are one of the main communication channels underlying bacteria–host crosstalk. One possible missing link implicated in the gut-immune-brain axis in stroke could be metabolites that are exclusively produced by bacteria.

Metabolite products of the essential aromatic amino acid tryptophan are known to modulate immune cell function through the aryl hydrocarbon receptor (AHR) which is expressed in both DC and T-cells (Lamas et al. 2018). Some bacteria members are capable of catabolizing tryptophan in the gut into ligands of AHR. In mice, activation of AHR by microbiota-derived indoles promotes intestinal homeostasis through induction of interleukin-22 in immune cells (Zelante et al. 2013). Activation of AHR in astrocytes by tryptophan metabolites drives neuroprotection in a mouse model of multiple sclerosis (Rothhammer et al. 2016). However, the protective effect seems to be ligand depend, as other tryptophan metabolites have been reported to aggravate the disease course (Veldhoen et al. 2008). Interestingly, the activity of kynurenine pathway responsible for tryptophan endogenous catabolism is upregulated following ischemic stroke and has been associated with the inflammatory response and a worsened outcomes (Brouns et al. 2010). Accordingly, pharmacological and genetic blockade of the AHR receptor improved stroke recovery (Chen et al. 2019). These findings suggest a pivotal role for tryptophan-derived compounds in stroke. Yet the immunomodulator effect of the microbiota-derived tryptophan metabolites in ischemic injury remains to be elucidated.

Short chain fatty acids (SCFA) are bioproducts of bacterial fermentation with immunomodulatory roles: they are able to directly induce T-cell differentiation into effector and regulatory cells depending on the immune context (Park et al. 2015). Strikingly, levels of plasma SCFA were significantly reduced after stroke in mice, and this was associated with a worsened outcome (Sadler et al. 2020). SCFA supplementation in mice prior to stroke improved behavioral recovery, modified cortical network connectivity, and changed histological markers of synaptic plasticity, which was associated with improved long-term stroke outcomes. These effects were correlated with modification of microglial morphology towards a homeostatic-like state and a reduction in brain-invading lymphocytes (Sadler et al. 2020). In another study, they observed that oral gavage of SCFA-producing bacteria and inulin, a bacterial substrate for SCFA production, diminished neurological deficits and improved depressive-like behaviors following stroke in aged mice in comparison to young mice. Moreover, these changes were accompanied with a reduced percentage of IL-17 + γδT cells in the ischemic brain (Lee et al. 2020), but whether SCFAs directly influence T-cell polarization, and migration was not addressed. Surprisingly, the volume of the infarct was not affected by SCFA interventions in both studies. All these evidences indicate that the microbial metabolite SCFAs play an important role in post-stroke recovery and may be involved in the immunomodulatory role exerted by the gut microbiota after stroke (Fig. 1).

## Conclusion

Here, we summarized the current findings on how the microbiota composition affects stroke outcome by modulating the immune response. The changes in gut microbiota following ischemic injury induces a predominantly pro-inflammatory T-cell response which is associated with greater infarct volume and worsened outcome. Because the gut microbiota is a very complex ecosystem, influenced by the environment and the host, with redundant functions and synergic relationships, identifying the functional microbial signature responsible for the neuroprotective effect could be a better approach rather than identifying specific bacterial species. Especially, since the composition of the gut microbiota substantially differs at deeper taxonomic level between humans and mice, it would be a better strategy to investigate the microbiome metabolomic profile for experimental research to be translated into clinics. However, to the best of our knowledge, there are no experimental data yet linking causality between specific microbiota-synthesized compounds and immune responses following brain damage. Further work addressing the molecular players involved in the crosstalk between commensal bacteria and the immune system after stroke would be necessary for successfully applying these findings in stroke patients.
